# Fluorescent homooxacalixarenes: recent applications in supramolecular systems

**DOI:** 10.3389/fchem.2023.1258026

**Published:** 2023-10-06

**Authors:** Paula M. Marcos, Mário N. Berberan-Santos

**Affiliations:** ^1^ Centro de Química Estrutural, Institute of Molecular Sciences, Faculdade de Ciências, Universidade de Lisboa, Lisboa, Portugal; ^2^ Faculdade de Farmácia da Universidade de Lisboa, Lisboa, Portugal; ^3^ IBB-Institute for Bioengineering and Biosciences and Associate Laboratory for Health and Bioeconomy, Instituto Superior Técnico, Universidade de Lisboa, Lisboa, Portugal

**Keywords:** homooxacalixarenes, supramolecular systems, fluorescence sensing, TADF, fullerene C_70_, metal cations, anions and ion pairs, explosives

## Abstract

This review covers recent advances (from 2006 to date) in supramolecular systems based on fluorescent homooxacalixarenes, namely hexahomotrioxacalix[3]arenes, dihomooxacalix[4]arenes and tetrahomodioxacalix[4]arenes, focusing on fluorescence sensing using their intrinsic fluorescence (built-in mesitol-like groups) or the extrinsic fluorescence of organic fluorophores, either covalently linked to the calixarenes or forming supramolecular complexes with them. Sensing applications of ions, ion pairs and neutral molecules are discussed, as well as the potential measurement of temperature based on thermally activated delayed fluorescence.

## Introduction

Supramolecular chemistry, first defined by J. M. Lehn as “chemistry beyond the molecule” (Lehn, 1995), focuses on the study of weak and reversible noncovalent interactions, such as hydrogen bonding and π-effects, present in molecular recognition and self-assembly processes (Huang and Anslyn, 2015; Amabilino and Gale, 2017). One of the main challenges in supramolecular chemistry is the construction of unique structures able to respond to external stimuli by using building blocks connected owing to intermolecular forces. Numerous applications in various areas, such as sensing, catalysis, molecular machines and drug delivery have been developed with these systems.

Host-guest chemistry based on macrocyclic compounds continues to be an important support of supramolecular chemistry. The ability of macrocycles, in particular calixarenes (Gutsche, 2008), to form host-guest complexes has been widely investigated. These phenol-based cyclic oligomers represent the third generation of supramolecular hosts and have attracted great interest over the past three decades (Neri et al., 2016). The availability of calixarenes, along with the easy functionalization at the upper or lower rims affords obtaining a large variety of derivatives, these being attractive building blocks for creating systems of increasing complexity and able to perform specific supramolecular functions.

Variations can be introduced into the calixarene skeleton. This is made possible by adding heteroatoms between the methylene bridges or replacing them entirely, to give the heteracalixarene class. Homooxacalixarenes, in particular, are calixarene analogues in which the CH_2_ bridges are partly or completely replaced by CH_2_OCH_2_ groups (Marcos, 2016). The incorporation of oxygen atoms and the higher conformational mobility brought by the dimethyleneoxa bridge modify the host-guest properties of these compounds (as compared to their all-carbon analogues), providing suitable binding sites for various species. Owing to this structural versatility, homooxacalixarenes have been used as receptors for different types of guests. For example, the *C*_3_ symmetry of the hexahomotrioxacalix[3]arenes (I), often encountered in nature but relatively rare in synthetic host molecules, makes them useful binders for guests with complementary binding requirements, as ammonium cations, species present in compounds of biological interest (Cottet et al., 2012).

The host-guest binding process is generally monitored by NMR, UV-Vis absorption or fluorescence spectroscopies. Among these analytical techniques, fluorescence spectroscopy, due to its high sensitivity, simplicity and real-time monitoring ability, is a very interesting method for the quantitative determination of ions and other analytes. Very low (sub-μM) concentrations, unattainable by NMR and UV-Vis absorption, can be accurately measured. Lately, fluorescent sensors based on calixarenes have been extensively used in various applications, namely in the detection of biologically and environmentally relevant cations and anions (Kumar et al., 2016; Kumar et al., 2019). A calixarene ion sensor, for fluorimetric assessment, must contain two units: an ionophore (binding unit) and a fluorophore (recognition unit). The first should bind selectively the ion by forming a host-guest complex, while the second signals the binding. Depending on the case, this response can be studied by steady-state fluorescence (quenching or enhancement, spectral changes) and/or by time-resolved fluorescence (lifetime constancy or variation) (Valeur and Berberan-Santos, 2012a). Although not widely recognised, calixarenes have a built-in fluorophore, and hence intrinsic fluorescence. However, this emission occurs in the UV and is not always responsive to guests. For this reason, various fluorophores, such as naphthalene, anthracene, pyrene, dansyl, coumarin, quinoline and naphthalimide have been covalently linked to the calixarene framework, leading to fluorescence probes for the recognition of different types of analytes (Kumar et al., 2019). The fluorophore can also bind to the calixarene cavity by supramolecular interactions only. In this case it is in fact the guest, but a guest that can be used to sense a third species, e.g. oxygen, which does not require binding. The role of the calixarene in this situation is that of a convenient matrix.

This review presents recent advances (from 2006 to date) in supramolecular systems based on fluorescent homooxacalixarenes, namely hexahomotrioxacalix[3]arenes (I), dihomooxacalix[4]-arenes (II) and tetrahomodioxacalix[4]arenes (III), focusing mainly on ion sensing applications.


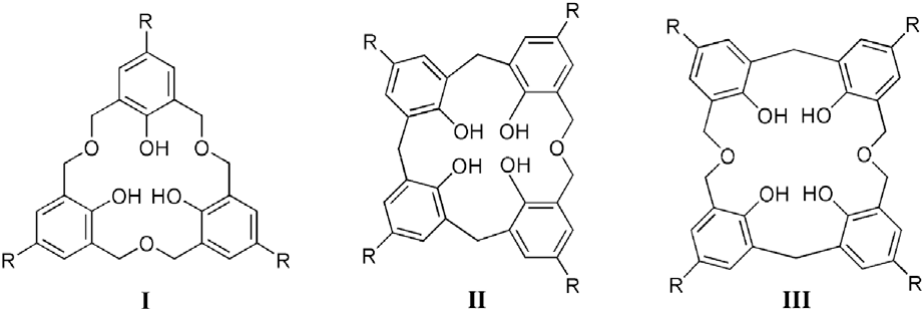


## Intrinsic fluorescence and supramolecular interactions

### Photophysical properties and intrinsic fluorescence

As happens with calixarenes, the intrinsic photophysical properties of homooxacalixarenes result from the skeletal benzenoid rings (2,4,6-trisubstituted phenol or phenoxyalkane moieties). Owing to the saturated nature of the bridges (methylene or dimethyleneoxa) no through-bond interaction between the aromatic moieties is possible. π-π stacking is also avoided owing to conformational constraints. In this way, the calixarenes behave as noninteracting sets of substituted phenols or phenol ethers (apart from a possible long-range energy homotransfer, to be discussed below).

**FIGURE 1:**
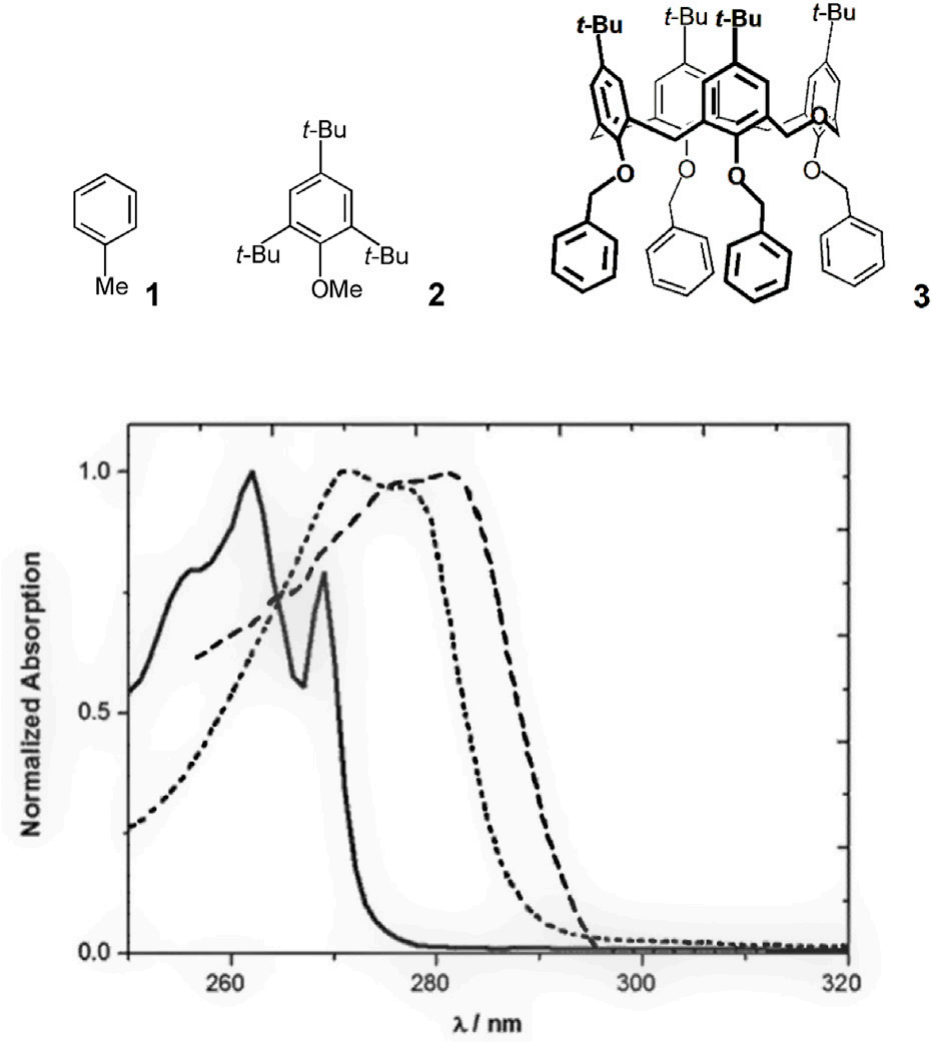
Normalized absorption spectra of **1** (solid line), **2** (dotted line) and **3** (dashed line) in MeCN (Miranda et al., 2017).

The properties of a typical homooxacalixarene, the *p-tert*-butyldihomooxacalix[4]arene tetrabenzyl ether **3**, are described. The electronic absorption spectra of **1** and model compound **2** in acetonitrile are shown in Figure 1, along with that of **3** (Miranda et al., 2017). All three absorb in the near ultraviolet. Similar spectra are observed in methylcyclohexane (MCH) and 1:1 MeOH/EtOH solutions. The S_0_ ← S_1_ ( $\pi ,\pi^{*}$ ) band in the absorption spectrum of **1** at room temperature is well characterized (Ginsburg et al., 1946; Burton, et al., 1968). It extends from about 280 nm to 240 nm featuring a few dominant vibronic bands with the strongest transition at 271 nm, corresponding to the (0,0)-band. The S_0_ ← S_1_ oscillator strength is relatively small, although the (0,0)-transition is significantly stronger than in the parent compound, benzene. At shorter wavelengths the spectrum shows less vibronic structure in relation with the more closely spaced transitions to higher excited singlet levels. The spectra of compounds **2** and **3** are red shifted with respect to that of toluene owing to the effect of additional substituents in the aromatic ring. Comparison of **2** and **3** allows concluding that the alkoxy group effect dominates the calixarene absorption in the long wavelength region (280–295 nm) (Miranda et al., 2017).

**FIGURE 2:**
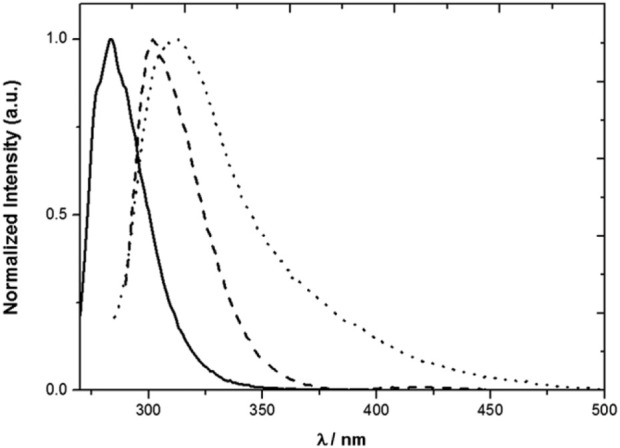
Normalized emission spectra of **1** (solid line), **2** (dotted line) and **3** (dashed line) in MeCN. *λ*_exc_ = 262 nm (**1**), 276 nm (**2**) and 280 nm (**3**) (Miranda et al., 2017).

The model compounds exhibit emission spectra in the near ultraviolet (**1** and **2**) also tailing in the visible in the case of **2** (Figure 2). The spectrum of **1** in MeCN at room temperature extends from roughly 260 nm–340 nm with a maximum at 285 nm, when excited at 262 nm. The maxima of the emission spectra of compounds **2** and **3** almost coincide and are red shifted with respect to the emission spectrum of toluene, as expected from the dominant contribution of the mesitol-like substituents.

The photophysical properties of **3** and of toluene and model compound **2** are collected in Table 1. It is seen that **2** and **3** are less fluorescent than **1**, and that **3**, although more fluorescent than **2**, displays a significantly shorter average fluorescence lifetime, implying a higher radiative decay rate constant (Miranda et al., 2017).

**TABLE 1:** Photophysical properties of compounds **1**, **2** and **3** in MeCN (Miranda et al., 2017).

	*λ*_max,abs_ (nm)	*λ*_max,em_ (nm)	*τ*_F_ (ns)	*Ф*_F_ ^a^
**1**	262	285	4.90	0.020
**2**	271/276	313	3.90	0.0091
**3**	280	305	0.94	0.018

^a^ Against **1** in degassed cyclohexane, *Ф*_F_ = 0.14 (Quina and Carroll, 1976).

**FIGURE 3:**
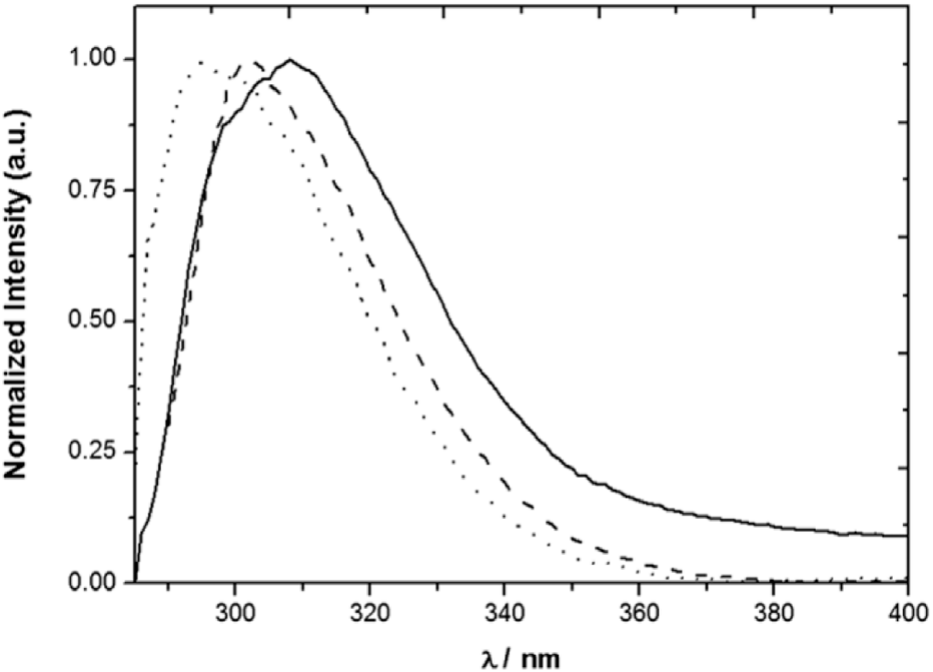
Normalized emission spectra of **3** in MCH (solid line), MeCN (dashed line) and 1:1 MeOH/EtOH (dotted line) solutions (Miranda et al., 2017).

The polarity of the medium affects both the fluorescence decay of compound **3** and its fluorescence spectrum (Figure 3) (Miranda et al., 2017). The fluorescence lifetime increases with the polarity of the solvent, going from 0.4 ns in an aprotic medium (MCH) to 1.4 ns in a polar medium (1:1 EtOH/MeOH). The fluorescence spectrum of **3** maintains the overall shape, but redshifts as the result of the increase in the solvent polarity, with maxima at 295 nm in MCH and 308 nm in 1:1 EtOH/MeOH. In contrast, the photophysical properties of model compound **2** are nearly insensitive to the solvent. Conformational changes in **3** may therefore be responsible for the polarity dependence observed for its emission.

### Homooxacalixarenes as high temperature matrices for TADF and temperature sensing

Thermally activated delayed fluorescence (TADF) is a specific type of fluorescence reflecting both singlet and triplet features: the emission spectrum is identical to that of the normal (also called prompt) fluorescence, whereas the lifetime is usually long and close to that of phosphorescence (Valeur and Berberan-Santos, 2012b). In the TADF mechanism, after the initial radiative transition S_n_ ← S_0_ and once the S_1_ state is reached, intersystem crossing (ISC) S_1_-T_1_ takes place, followed by a second ISC back to S_1_, T_1_-S_1_, from which fluorescence occurs. Note that the S_1_-T_1_-S_1_ cycle may repeat itself several times (Berberan-Santos and Baleizão, 2007), however the TADF mechanism is only possible for very low concentrations of molecular oxygen, otherwise the triplet state is quenched and the cycle is interrupted (Valeur and Berberan-Santos, 2012b). Not too low temperatures are also usually required, given that the reverse ISC is thermally activated. In some systems, the energy gap is very low and the temperature dependence is weak. Matrices capable of withstanding high temperatures and simultaneously not quenching TADF are still of great interest. Additionally, the usually strong temperature dependence of TADF allows designing temperature sensors and nanosensors based on this phenomenon (Baleizão et al., 2007).

Owing to its (currently still) potential application in commercial OLEDs (Organic Light Emitting Diodes), there was a surge of interest on TADF in the last decade, leading to the synthesis of hundreds of new molecules having this property (Gawale et al., 2023). Fullerenes, especially C_70_ display an extraordinary TADF efficiency, as shown by their singlet-triplet interconversion diagram (Palmeira and Berberan-Santos, 2017). Owing to the long lifetime of the triplet state (millisecond range) and broad TADF temperature range (from −60°C up to at least 100°C), C_70_ and derivatives have been applied in sensitive oxygen (in the ppmv range) and temperature optical sensors. In 2011, it was discovered the carbon-13 (C-13) isotope effect in luminescence (not previously observed in any molecule), and in particular in C_70_: the triplet lifetime of C-13 C_70_ is approximately the double of that of normal (C-12) C_70_. This implies that TADF efficiency is much higher in C-13 C_70_, making it the brightest fullerene (Baleizão and Berberan-Santos, 2011), and allowing its use in a highly sensitive optical oxygen sensor (ppbv range) (Kochmann et al., 2013). The temperature range of TADF in polymer matrices is of necessity limited by the maximum temperature at which they remain rigid (the glass transition temperature) or chemically stable, this being ca. 100°C for polystyrene. For work at temperatures significantly above 100°C (while retaining rigidity and chemical stability), new polymer matrices are required.

**FIGURE 4:**
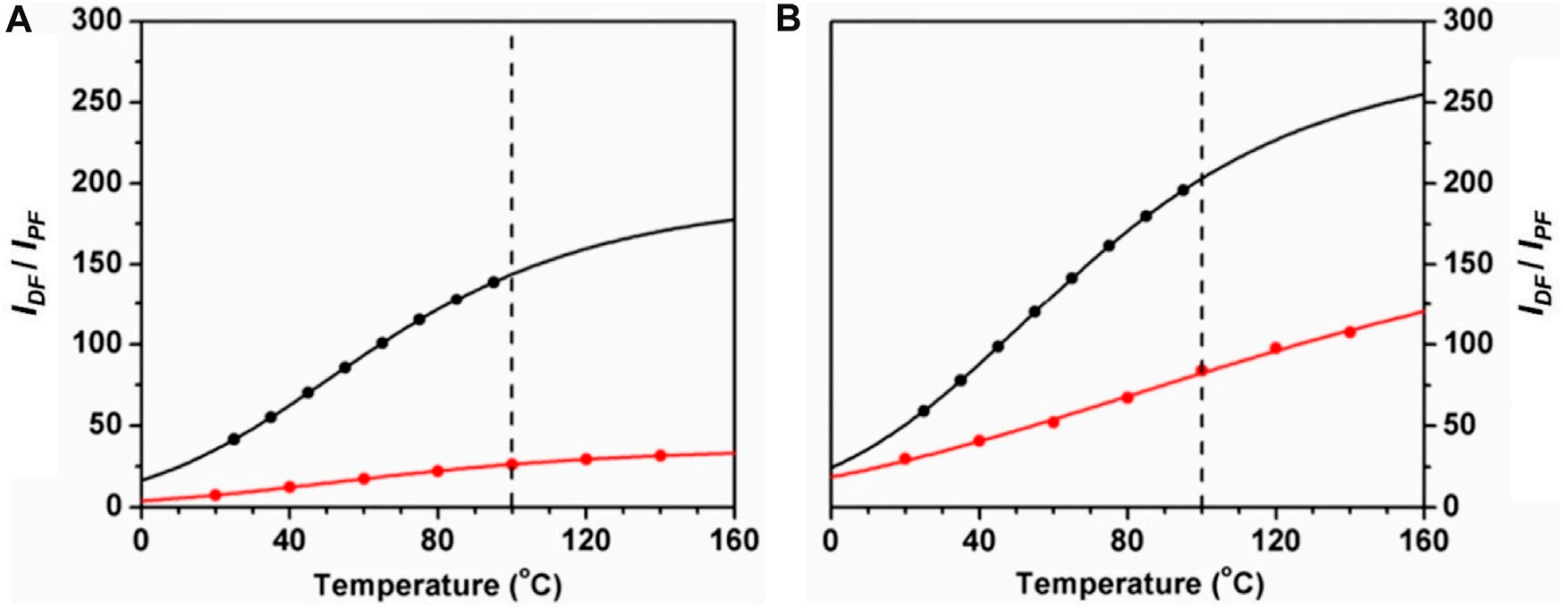
Delayed-to-prompt-fluorescence ratio *I*_*DF*_ */I*_*PF*_ versus temperature for ^12^C_70_ **(A)** and ^13^C_70_ **(B)** in dihomooxacalix[4]arene (red) and in PS (black). The lines correspond to theoretical fits, and the vertical dashed line is the upper temperature limit for PS (Palmeira et al., 2018).


Parent calixarenes (those with free hydroxyl groups at the lower rim) have a bowl shape cavity with internal π-electrons, which may indicate good host properties for nearly spherical guests with external π-electrons, such as fullerenes. These calixarenes are also characterized by high melting points, usually above 250°C. For this reason, *p-tert*-butyldihomooxacalix[4]arene (**4**) was chosen as a solid matrix for high temperature TADF measurements of normal and C-13 enriched fullerene C_70_. It was observed that significant TADF occurs up to at least 140°C (experimental set-up upper limit), as shown in Figure 4 (Palmeira et al., 2018).

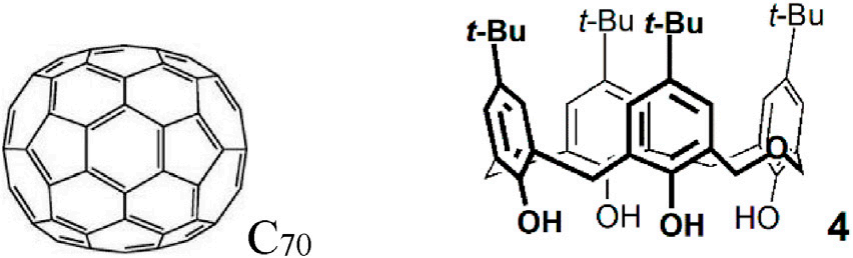


**FIGURE 5:**
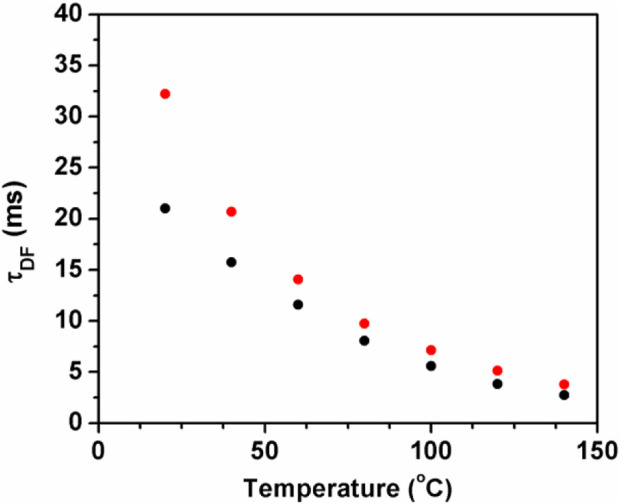
Delayed fluorescence lifetime versus temperature for ^12^C_70_ (black) and ^13^C_70_ **(**red**)** in the dihomooxacalix[4]arene matrix (Palmeira et al., 2018).

The delayed fluorescence lifetime was also measured as a function of temperature (Figure 5). These dependencies provide an absolute calibration for the measurement of temperature by means of delayed fluorescence (Baleizão et al., 2007). The results obtained open the possibility of covering temperatures up to 200°C or more.

### Electronic energy hopping (homo-FRET) and combined homo- and hetero-FRET

The close proximity (but off-contact) of the mesitol-like fluorophores in the homooxacalixarenes, with distances between neighbouring chromophores of the order of 0.5–0.7 nm, and the significant value of the Förster radius for FRET self-transfer (ca. 1.4 nm), allow the existence of fast and reversible homo-FRET in these systems (Miranda et al., 2021).

**FIGURE 6:**
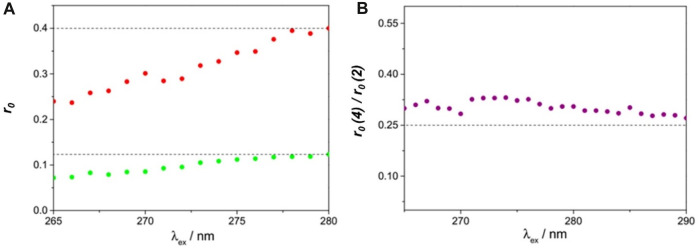
Fluorescence excitation anisotropy spectra of **4** (green) and **2** (red) in a Zeonex film at room temperature **(A)**, and the respective anisotropy ratio **(B)**, as a function of excitation wavelength. Slits were 8 nm for excitation and 14 nm for emission. λ_em_ = 306 nm (Miranda et al., 2021).

Evidence for pure homotransfer is obtained from fluorescence anisotropy measurements of both parent dihomooxacalix[4]arene **4** and model compound **2** in a rigid medium (Zeonex film). The respective excitation fluorescence anisotropy spectra are shown in Figure 6, as well as the ratio of the two anisotropies. The anisotropy r_0_(λ_exc_) of compound **2** (Figure 6) attains the value of 0.40 at the S_1_←S_0_ absorption onset (280 nm), corresponding thus to coincident absorption and emission transition dipole moments (^1^L_b_ band) (Valeur and Berberan-Santos, 2012a).

The excitation wavelength-dependence of the anisotropy along the first band shows a progressive decrease down to 0.24 at 260 nm, being compatible with a mixed polarisation, where the effect of the orthogonally polarised ^1^L_a_ band increases when decreasing the excitation wavelength. The fluorescence anisotropy excitation spectrum of **4** (Figure 6) follows the same trend, albeit with lower values, starting from 0.12 at 280 nm. The drop in anisotropy observed for this calixarene can be entirely ascribed to fast energy migration (FRET homotransfer). Given that there are four equivalent chromophores, with nearly isotropic and uncorrelated orientations, the anisotropy is expected to reduce to r_0_(λexc)/4 owing to FRET (Berberan-Santos et al., 1992). As seen in Figure 6, this is indeed borne out by the experiment, with a ratio of 0.30 ± 0.03 being obtained between 265 and 290 nm. The slight excess over 0.25 can be ascribed to a residual degree of orientational anisotropy and orientational correlation.

**FIGURE 7:**
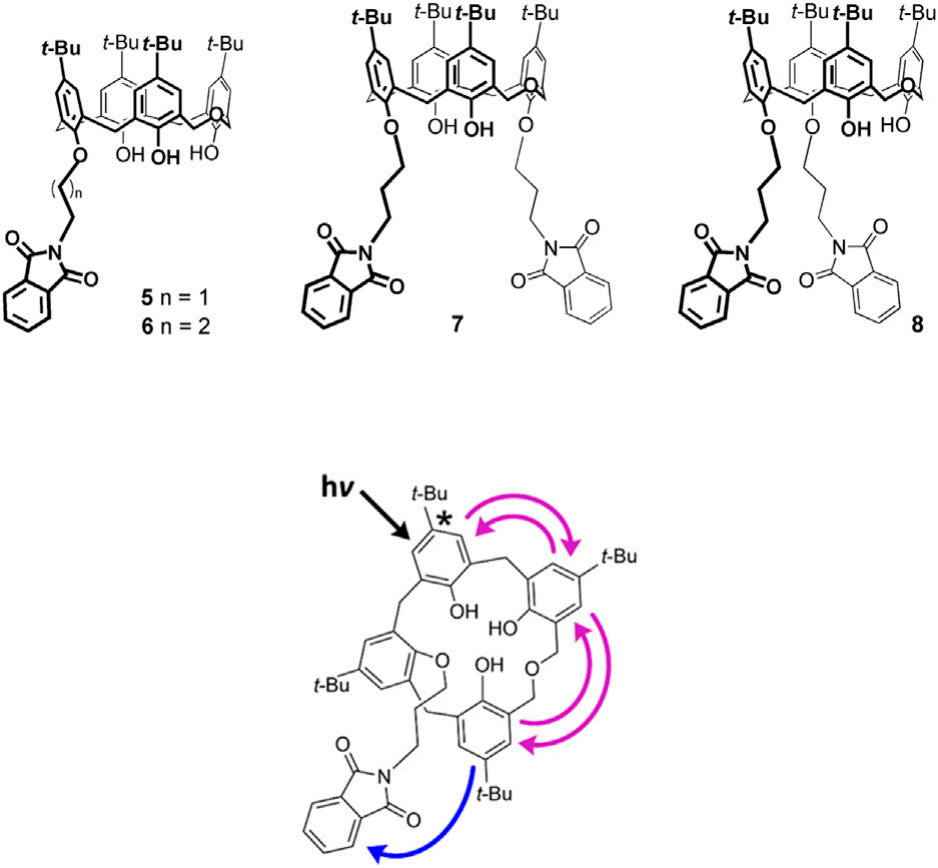
Schematic representation of homo-FRET and hetero-FRET in compound **6**. Upon excitation, homotransfer (reversible FRET) takes place within the calixarene and is followed by heterotransfer (irreversible FRET) from the calixarene to the phthalimide group (only one of many possible excitation outcomes is shown) (Miranda et al., 2021).

Homooxacalixarenes containing one or two covalently linked phtalimides (**5–8**) that absorb at longer wavelengths, open the possibility of homotransfer combined with heterotransfer, again of the FRET type, when the mesitol-like fluorophore is excited (Figure 7) (Miranda et al., 2021). It is indeed found that the fluorescence quantum yield decreases (by one or two orders of magnitude) in the case of compounds **5**, **7**, **8**. The two compounds bearing two acceptor phthalimides, **7** and **8**, display similar but lower donor fluorescence quantum yields when compared to the equivalent monophthalimide compound **6**, as expected. The monophthalimide **5** differs from all the others by the number of carbon atoms of the spacer attaching the phthalimide (C2 vs. C3). The lower fluorescence quantum yield of **5** (in fact, the lowest of all) is in accordance with the shortest (C2) chain, hence the shortest average donor-acceptor distance (down to 0.6 nm).

## Chemical sensing applications

### Metal cations

Fluorescent sensors based on hexahomotrioxacalix[3]arenes for transition and heavy metal cations have been reported mainly by Yamato and coworkers.

The cone sensor **9**, bearing a rhodamine amide fluorophore, was investigated towards several metal ions by UV-Vis and fluorescence spectroscopies (Wu et al., 2010). Strong colorimetric changes and significant fluorescent enhancement were observed upon the addition of Sb^3+^, Fe^3+^ and Ni^2+^ to **9**, while almost no response was shown with other cations, such as Fe^2+^, Cu^2+^, Co^2+^, Zn^2+^, Cd^2+^, Hg^2+^ and Ag^+^. This compound may therefore be used as a turn-on type fluorescent probe.


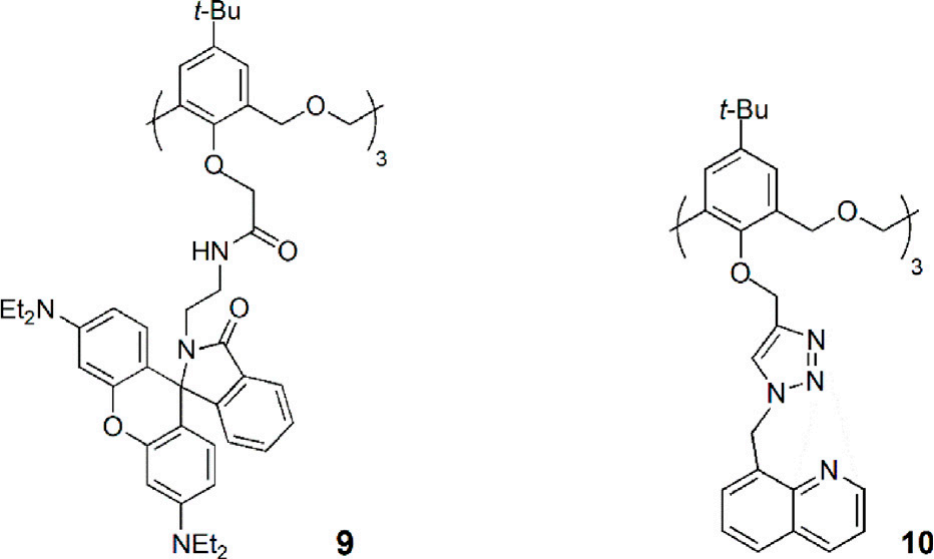


Recently, Yamato and coworkers reported the synthesis of a hexahomotrioxa quinoline-triazole derivative (**10**) by click chemistry and its high selectivity for Fe^3+^ with little interference from other metal cations, including alkali, transition and heavy metal ions (Wu et al., 2018). Chemosensor **10** presents a turn-on type fluorescent behaviour, with a detection limit to Fe^3+^ in the order of 10^–7^ M. **10** forms a 1:1 complex with Fe^3+^ and the sensing mechanism may be due to the inhibition of the PET process (photoinduced electron transfer) during **10**-Fe^3+^ binding.

Two cone hexahomotrioxa fluorescent sensors **11** and **12** containing pyrene-linked triazole moieties at the lower rim showed high affinity and selectivity for Pb^2+^ in comparison to other competitive metal ions tested (Ni et al., 2011a). The association constant of the 1:1 complex of **12**-Pb^2+^ is 2.60 × 10^5^ M^−1^ in MeCN/H_2_O/DMSO (1000:50:1), showing the potential of **12** to be used as a ratiometric fluorescent chemosensor for Pb^2+^ ions. The sensing mechanism is due to the pyrene moieties, which are appended to the calixarene skeleton by triazole groups to form a strong excimer. The result is the decrease of the excimer emission intensity and the consequent increase of the monomer emission intensity upon Pb^2+^ binding by the triazole rings.


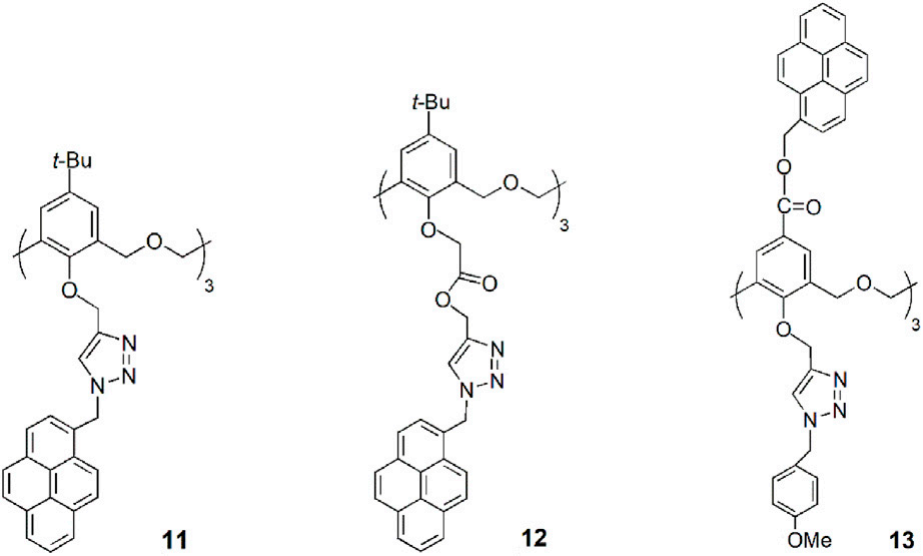


A new hexahomotrioxa chemosensor **13** bearing a fluorophore pyrene at the upper rim of the calixarene and an ionophore triazole unit at the lower rim was developed and showed different cation sensing behaviours (Wu et al., 2015). According to ^1^H NMR titration experiments, the transition and heavy metal ions Zn^2+^, Cu^2+^, Hg^2+^ and Pb^2+^ are bound by the three triazole moieties, resulting in a conformational change, that causes an increase of the monomer emission intensity and an excimer quenching, while in the case of Fe^3+^ binding, which takes place at the macrocycle cavity, the approach of the pyrene units produces an increase of the emission intensity of the excimer. The highest *K*_ass_ was obtained for the 1:1 complex of **13**-Pb^2+^ with the value of 6.88 × 10^5^ M^−1^ in MeCN. The authors believe that the calixarene scaffold acts as a spacer, preventing a significant metal cation-fluorophore interaction, thus preventing the heavy atom effect.

A novel hexahomotrioxa derivative **14** functionalized with pyrene-linked 1,2,3-triazole units at the lower rim of the calixarene and ethyl ester groups at the upper rim, was obtained as a fluorescent chemosensor for Zn^2+^ in a mixed aqueous media (*K*_ass_ = 7.05 × 10^4^ M^−1^ in MeCN/H_2_O, 10:1, for the 1:1 complex) (Wu et al., 2016a). The monomer **M**, used as a reference compound and studied in the same conditions, showed no significant changes of its fluorescence spectrum upon addition of Zn^2+^ and other metal cations. These results indicated that Zn^2+^ selective recognition depends on the synergistic action of the three triazole units.


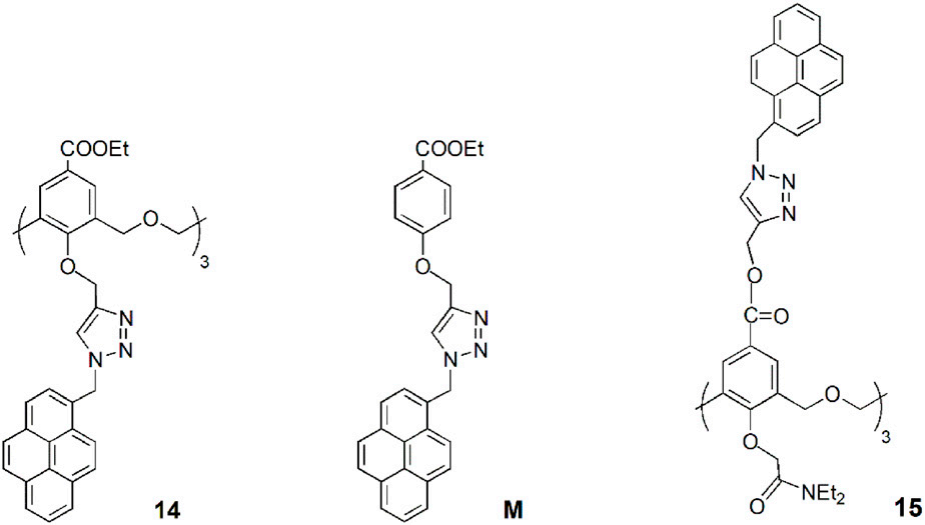


**FIGURE 8:**
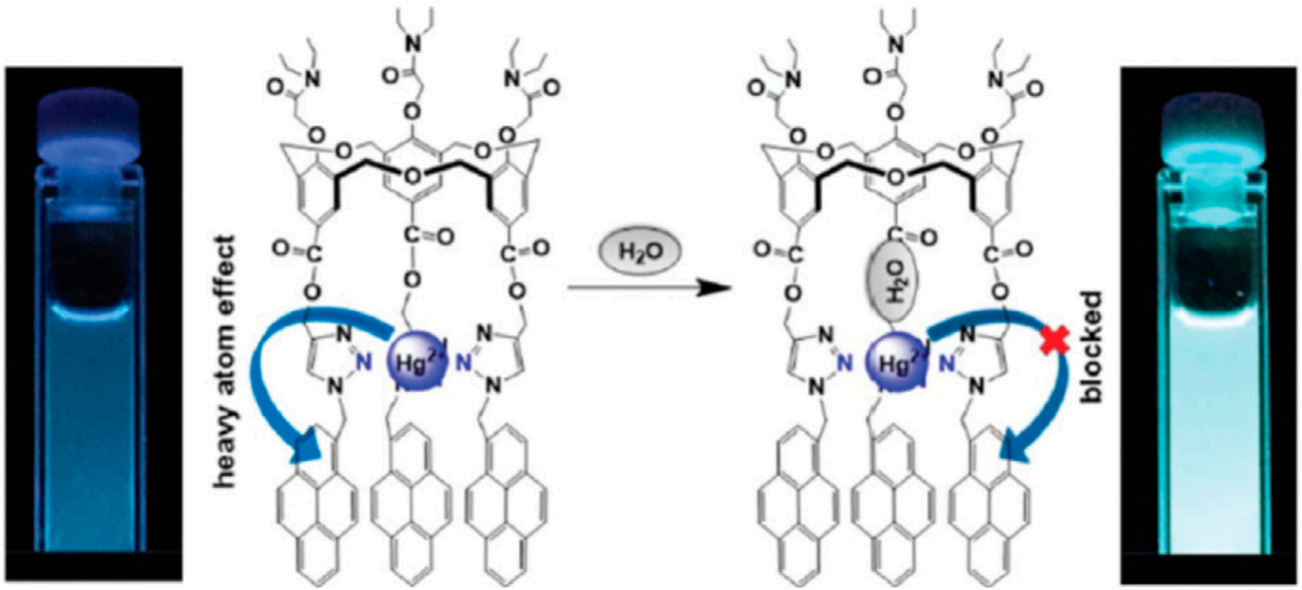
A possible mechanism for the heavy atom effect blocked by H_2_O in the **15**-Hg^2+^ complex. Reproduced from Wu et al., 2016b, with permission of The Royal Society of Chemistry.

Another pyrene-functionalized hexahomotrioxacalix[3]arene at the upper rim and bearing di-ethyl amide groups at the lower rim (**15**) exhibited a significant fluorescence quenching (up to 96%) upon addition of Hg^2+^ cations in MeCN (Wu et al., 2016b). Moreover, it was observed that the heavy atom effect of Hg^2+^ can be blocked by coordination of a water molecule, resulting in the regeneration of the fluorescence emission of the **15**-Hg^2+^ complex (Figure 8). Other polar solvents, such as methanol and ethanol, also showed this regeneration effect of the fluorescence emission of the complex. The inhibition of the heavy atom effect of Hg^2+^ and Cu^2+^ by water and other polar solvent molecules had already been reported for chemosensor **12** (Ni et al., 2014).

A new type of fluorescent sensor (**16**) containing triazole groups as the binding site and anthracene as the fluorogenic unit on the lower rim of a hexahomotrioxacalix[3]arene was obtained in the cone conformation (Jiang et al., 2016). Compound **16** showed a very high selectivity for Zn^2+^ and Cd^2+^, even in the presence of excess of competing metal ions, behaving as a fluorescence enhancement type chemosensor. ^1^H NMR experiments indicated that upon Zn^2+^ binding the macrocycle cavity changes from a *flattened-cone* to a more upright form, and the anthracene moieties move away from each other, reducing the π-π stacking and causing fluorescent enhancement. By other side, **16** presented as a fluorescence quenching type chemosensor toward Cu^2+^ and Fe^3+^ ions, due to the PET process or the heavy atom effect.


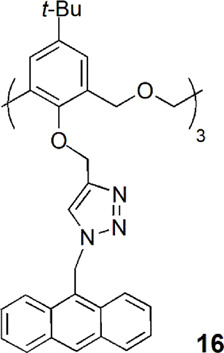


Besides the binding studies with hexahomotrioxacalix[3]arene derivatives, two reports of fluorescent sensors based on tetrahomodioxacalix[4]arenes for metal cation binding were also found in the literature since 2006.

J. S. Kim and coworkers obtained a series of 1,2-alternate tetrahomodioxa-*p*-phenylcalix[4]arenes tetra-(**17)** and di-substituted (**18**) with pyreneamide groups at the lower rim (Choi et al., 2006). In both cases, a selective decrease of the monomer and excimer fluorescence emission was observed upon Pb^2+^ complexation. The coordination of Pb^2+^ ion to the carbonyl groups of the amides induces a conformational change, which causes the disruption of the Py-Py* interaction. A reverse-PET and the heavy metal ion effect are the main factors for the monomer emission quenching. The di-substituted tetrahomodioxa receptor **18** also showed affinity for Ca^2+^. The binding of this cation causes an enhanced excimer and a decreasing monomer emission with ratiometric response. DFT calculations (B3LYP/3-21G* level) were also employed for both hosts and their complexes to better understand the fluorescence behaviour. The results showed that the HOMO-LUMO interaction between Py and Py* induces a strong excimer emission upon Ca^2+^ complexation, while such interaction is not observed upon Pb^2+^ binding.

In 2009, the previous authors developed a novel tetrahomodioxa derivative (**19**) bearing both naphthyl- and pyrenyl-amide groups at the lower rim, as a FRET-based fluorimetric sensor for Cu^2+^ cation (Jung et al., 2009). Intramolecular FRET was observed, from naphthalene emission to the pyrene absorption, binding Cu^2+^ ion selectively over other metal ions tested.


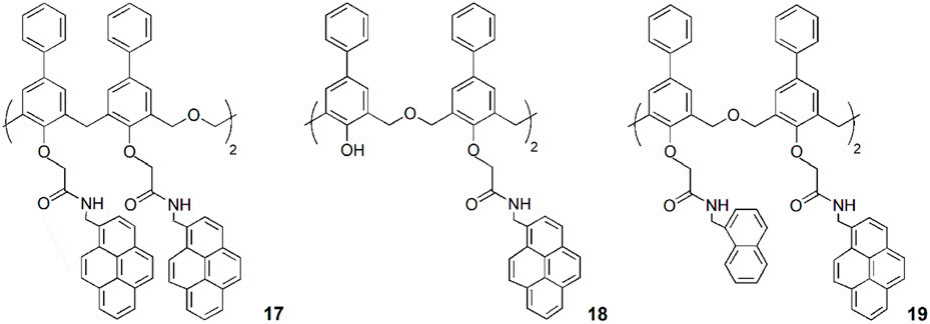


### Anions and ion pairs

Anion recognition by calixarenes continues to attract much attention due to the essential roles played by anions in numerous biological and environmental systems (Busschaert et al., 2015). Some of these hosts are able to simultaneously bind both ions of a given salt (He et al., 2019; McConnell et al., 2020). This ion pair recognition can be a cooperative process, as the binding of one ion enhances the binding of the other.

Yamato and coworkers reported that hexahomotrioxa **12** also exhibited an increased fluorescence intensity upon addition of a large amount of Zn^2+^, forming a 1:1 complex with a *K*_ass_ of 9.51 × 10^4^ M^−1^ in organic solvents (CH_3_CN/CH_2_Cl_2_, 1000:1), but the monomer emission of this **12**-Zn^2+^ complex was strongly inhibited in an organic/aqueous solvent mixture (Ni et al., 2011a). Inspired by those results, the fluorescent behaviour of the **12**-Zn^2+^ complex toward several anions was studied (Ni et al., 2011b). Authors observed a 67% enhancement of the excimer fluorescence intensity upon addition of 40 equiv of H_2_PO_4_^−^ compared to that of **12**-Zn^2+^ complex alone. Other anions tested induced slight monomer and excimer emission quenching only. Receptor **12** seems to have the capacity to sense both Zn^2+^ and H_2_PO_4_^−^ ions with different optical signals. In addition, the fluorescence changes by the inputs of Zn^2+^ and H_2_PO_4_^−^ ions were used to develop an R-S latch logic circuit at the molecular level.

A new upper rim pyrene-hexahomotrioxa sensor (**20**) was developed, which showed a pronounced increase in the monomer emission and a similar decrease in the excimer emission upon addition of Cu^2+^ ions (Jin et al., 2015). No significant spectral changes were observed upon addition of other metal cations, except for Zn^2+^ and Cd^2+^. The **20**-Cu^2+^ complex exhibited a high sensitive response to the latter cations, suggesting that it could be used as a sensor for Zn^2+^ and Cd^2+^. Due to the high affinity of F^−^ anion toward Cu^2+^, the complex **20**-Cu^2+^ was also investigated as a potential indirect sensor for F^−^ detection. The authors concluded that **20**-Cu^2+^ complex can recognize F^−^ via anion-induced demetallation and the presence of Zn^2+^ and Cd^2+^ leads to a pronounced fluorescence enhancement of the complex through a synergistic effect. Based on the fluorescent behaviour of sensor **20**, a combinational logic gate was constructed, which mimics a set of molecular traffic signals.

Another hexahomotrioxa derivative bearing pyrenyl amide residues at the lower rim (**21**) was synthesised by Yamato group (Xie et al., 2020). This chemosensor showed a high affinity and selectivity toward I^−^ anion, exhibiting a significant quenching of the emission intensity and little interference from other competitive anions.


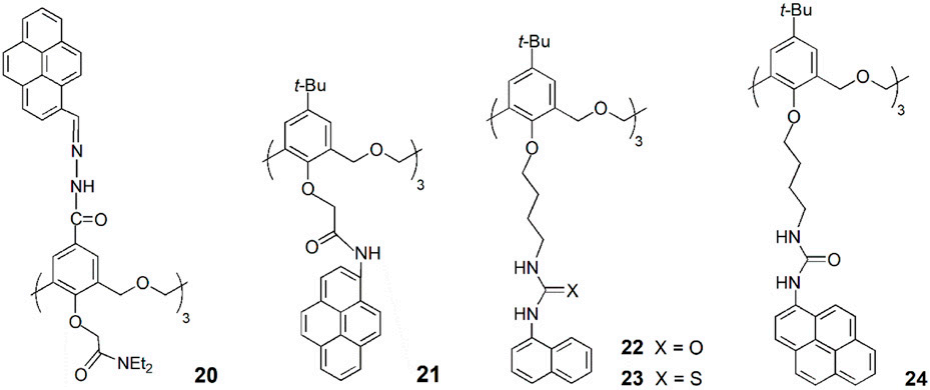


The anion binding properties of three fluorescent (thio)ureido-hexahomotrioxa receptors bearing naphthyl or pyrenyl residues at the lower rim and presenting the partial cone conformation (**22–24**) were investigated by Marcos and coworkers (Miranda et al., 2022). The results obtained by NMR, UV-Vis absorption and fluorescence titrations showed that the association constants for the 1:1 complexes increase with the anion basicity in general for all the receptors. The halide F^−^ and the carboxylates AcO^−^ and BzO^−^ were the best bound anions and the pyrenyl urea derivative (**24**) the more efficient receptor (log *K*_ass_ = 3.31, 3.42 and 3.45, respectively, determined by NMR in CDCl_3_). Thermodynamics data were obtained for **22** with F^−^ and AcO^−^ anions by UV-Vis and fluorescence in acetonitrile and indicated that the binding process is entropy-driven. The computational studies carried out pointed to the critical role of the solvent on the anion-receptor association. Compounds **22** and **24** were also tested as ditopic receptors in the recognition of *n*-alkylammonium chlorides, but at low temperature (233 K in CDCl_3_) the association/dissociation process was still fast on the NMR time scale.

Taking advantage of the intrinsic fluorescence of dihomooxacalix[4]arenes, some derivatives have been investigated concerning their anion complexation properties, mainly by Marcos, Berberan-Santos and coworkers. The binding affinity of dihomooxa phenylureas **25** and **26** towards NO_3_^−^ and HSO_4_^−^ anions was determined by steady-state fluorescence (Miranda et al., 2017). An increase of the emission intensity was observed in all cases. Both receptors showed slightly higher association constants for HSO_4_^−^ and phenylurea **25** exhibited a more effective behaviour as anion receptor than **26a**. The NO_3_^−^ and HSO_4_^−^ binding constants were also determined by fluorescence in CH_2_Cl_2_ with the electron-withdrawing receptor **26b** (Miranda et al., 2019). As expected, the constants were higher than those obtained for **26a**, but followed the same trend.


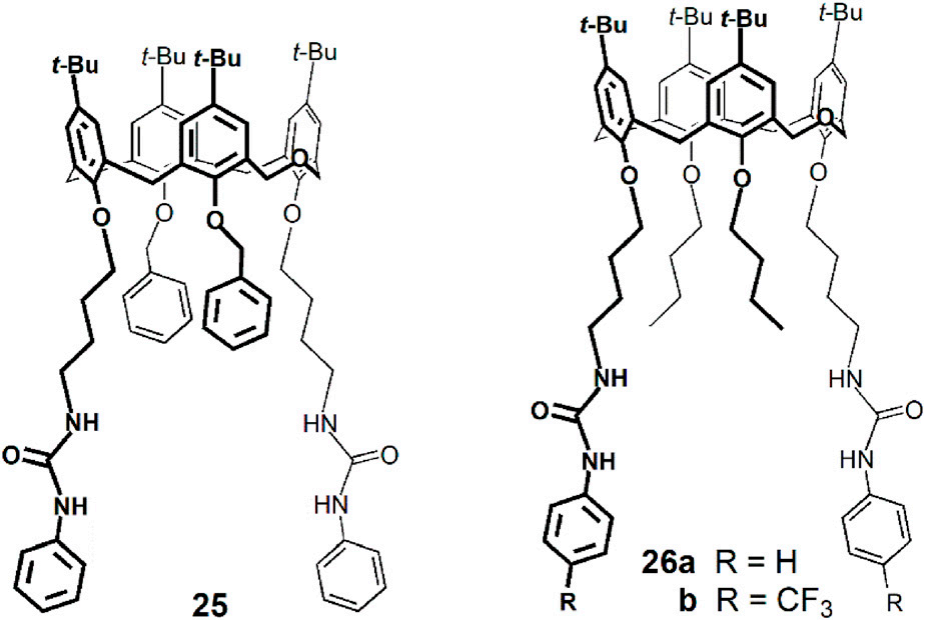


Fluorescent dihomooxa receptors containing two naphthyl urea moieties in distal (**27**) or proximal (**28**) positions at the lower rim were obtained in the cone conformation (Miranda et al., 2020). Fluorescence titrations showed an increase of the emission intensity upon addition of F^−^, AcO^−^ and H_2_PO_4_^−^anions, while a quenching of the fluorescence intensity was observed in the case of Cl^−^ and BzO^−^. The fluorescence lifetimes of the 1:1 complexes in the case of Cl^−^ anion showed a moderate decrease compared to those of the free receptors, while the quantum yields dropped significantly (Table 2). This indicates the existence of a marked static quenching in the complexes. The quenching was similar with respect to both intensity and lifetimes in the case of BzO^−^, arising mainly from an increase in the nonradiative decay constant in the complex, where aromatic moieties of receptor and anion appear to interact. The highest association constants determined by fluorescence in CH_2_Cl_2_ were found for F^−^, AcO^−^ and BzO^−^ anions, and proximal naphthyl urea **28** was a slightly better receptor than distal **27**. These results were corroborated by DFT calculations (B3LYP/6-31G(d,p) level).

**TABLE 2:** Photophysical properties of Naphureas **27** and **28** in CH_2_Cl_2_ at 25°C (Miranda et al., 2020).

	*λ*_max,abs_ (nm)	*ε* (M^–1^ cm^–1^)	*λ*_max,em_ (nm)	*τ*_*F*_ (ns)	*Ф*_F_ ^a^	*k*_r_ (ns^–1^)	*k*_nr_ (ns^–1^)
**27**	283	$1.5\times 10^{4}$	382	8.93	0.31	0.035	0.077
**27 +** Cl^−^	303	$2.4\times 10^{4}$	382	6.26	0.085	─	─
**27** + BzO^–^	303	$2.0\times 10^{4}$	382	1.98	0.048	0.024	0.48
**28**	282	$1.8\times 10^{4}$	379	7.57	0.26	0.034	0.098
**28** + Cl^−^	302	$2.3\times 10^{4}$	379	6.63	0.16	─	─
**28** + BzO^–^	302	$1.7\times 10^{4}$	379	3.27	0.078	0.024	0.28

^a^ Against quinine sulfate *Ф*_F_ = 0.546 in H_2_SO_4_ 0.5 M.

2,3-Ethylene-bridged dihomooxa derivative **29** was obtained and its binding affinity towards several anions assessed by fluorescence titrations (An et al., 2018). The results indicated a significant decrease of the fluorescence intensity upon addition of I^−^, and very small effects in the presence of other anions. The same authors also investigated the binding properties of some dihomooxa derivatives bearing thiourea residues at the lower rim, but the emission profiles of these compounds were not affected upon addition of tetrabutylammonium halides (Zhao et al., 2019).


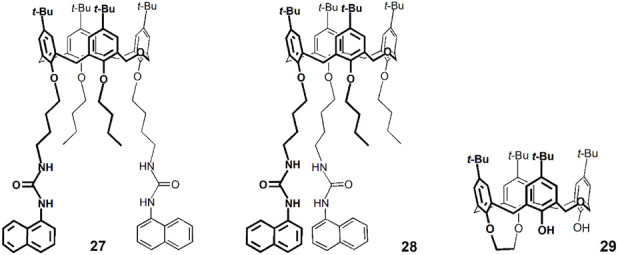


### Other guest species

Besides the ion sensing studies of fluorescent homooxacalixarenes described previously, the recognition of neutral species by these receptors have also been reported.

Nitroaromatic compounds (NACs), such as trinitrotoluene (TNT), dinitrotoluene (DNT) and trinitrophenol (TNP), are common explosives used for military purposes and are the principal components of unexploded landmines. They are also considered environmental pollutants. The detection of these and other explosives is thus a major task in the fight against terrorism and in homeland security.

Hexahomotrioxa compounds **11** and **14** were tested in the detection of several NACs by Yamato group (Wu et al., 2016c). The fluorescence emission intensity of **11** and **14** decreased markedly upon addition of increasing amounts of TNP, indicating strong interaction between the hosts and the guest. The Stern–Volmer plots were linear at lower concentrations (up to 100 µM) and the corresponding constants were calculated to be 2.2 × 10^4^ and 1.0 × 10^4^ M^−1^ in acetonitrile for **11** and **14**, respectively. However, no correction to these values owing to a likely absorption of the excitation radiation by TNP is mentioned in the article.

**FIGURE 9:**
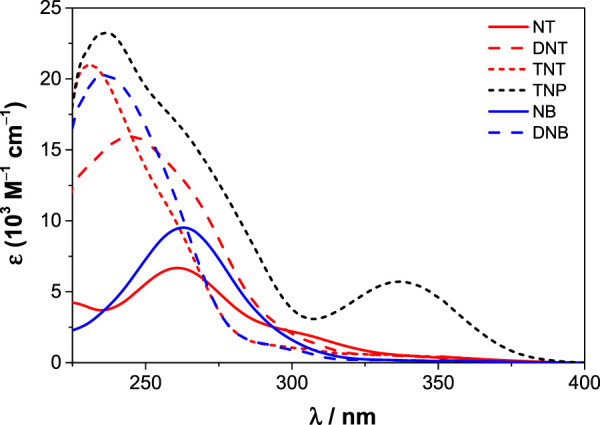
Molar absorption coefficients of nitroaromatic compounds (NT, DNT, TNT, TNP, NB and DNB) in CH_2_Cl_2_ (Miranda et al., 2023).

Two other hexahomotrioxa derivatives (**22** and **24**) were also evaluated in the recognition of some NACs (Miranda et al., 2023). A decrease in the fluorescence intensity was observed upon addition of all the NACs to **22** in CH_2_Cl_2_ and also in MeCN, and upon addition of TNP to **24** in CH_2_Cl_2_. As all the NACs studied significantly absorb at 300 nm and TNP absorbs also at 340 nm (*λ*_exc_ used in the case of **22** and **24**, respectively) (Figure 9), it was mandatory to correct the absorption of the NACs at the excitation wavelength (inner filter effect) to be able to calculate correct Stern–Volmer constants (*K*_SV_). The results obtained showed in general no significant interaction between both derivatives and the NACs. DFT calculations and MD simulations were also performed and corroborated those results. This study clearly demonstrates the importance of considering the inner filter effect (both excitation and emission) in these systems. Greatly overestimated association constants can be obtained if no correction is applied. These effects are often overlooked or completely neglected in complexation studies based on fluorescence.

**FIGURE 10:**
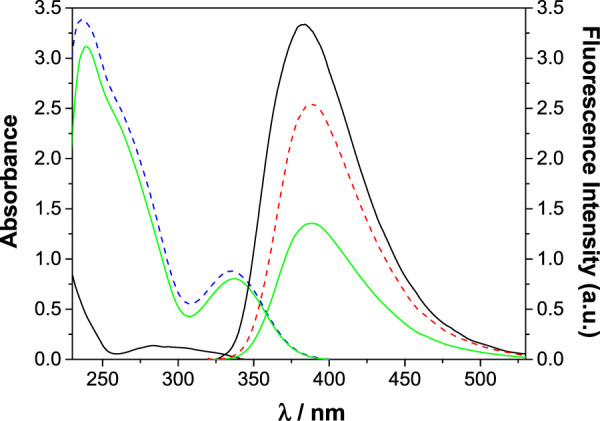
Absorption and fluorescence in CH_2_Cl_2_. Left side (short wavelengths): absorption spectra of **27** (10 μM) alone (black) and in the presence of 15 equiv of TNP (green). The absorption spectrum of 15 equiv of TNP is also shown (dashed blue). Right side (long wavelengths): fluorescence spectra ( $\lambda_{\text{exc}}=$ 300 nm) of **27** (10 μM) alone (black) and in the presence of 15 equiv of TNP, before (green) and after (dashed red) correction for the inner filter effect (Miranda et al., 2023).

The potential of dihomooxa naphthylurea **27** as a chemosensor to detect nitroaromatic explosives is currently in investigation by fluorescence studies. Preliminary results (Miranda et al., 2023) showed that upon addition of TNP to **27** in CH_2_Cl_2_ a significant quenching of the fluorescence intensity was observed after the excitation inner filter correction (Figure 10). Moreover, the quenching is static, corresponding to the formation of an association complex. The true and the apparent (uncorrected) *K*_SV_ for a 1:1 complex were determined as 3000 and 9400 M^−1^, respectively, stressing again the importance of taking into account the inner filter effect.

A new functionalized dihomooxa derivative (**30**) was obtained through a muiti-component Ugi reaction and used in the preparation of a soft gel (Guo et al., 2020). According to the authors, this supramolecular gel can include small molecules as drugs and then release them in a sustained way in water. The organic dye alizarin red S (**31**) was used as a model compound, and it was incorporated in the soft material forming a fluorescent gel. This gel was immersed in water, which was changed every 12 h, and the fluorescence intensity of the solution remained approximately constant after ten cycles.


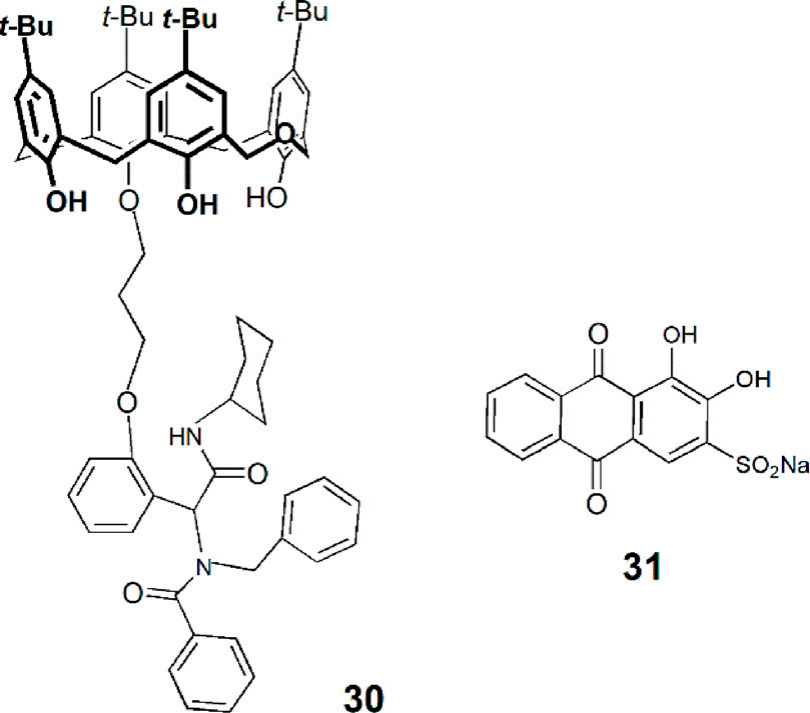


## Outlook

Host-guest chemistry based on calixarenes has been extensively investigated. Homooxacalixarenes, which are calixarene analogues in which the CH_2_ bridges are partly or completely replaced by CH_2_OCH_2_ groups, have been much less studied. However, their host-guest properties are different to those of their all-carbon analogues, providing a more suitable binding in some cases. Homooxacalixarenes have indeed been used as molecular platforms in the development of receptors for different types of guests.

Monitoring the host-guest binding process by fluorescence spectroscopy is advantageous, owing to the high sensitivity, simplicity, and real-time monitoring ability of this technique. Furthermore, very low (sub-μM) concentrations, unattainable by NMR and UV-Vis absorption, can be accurately measured. Lately, and as reviewed here, fluorescent sensors based on homooxacalixarenes have been used in various applications, namely in the detection of biologically and environmentally relevant cations and anions, as well as for the monitoring of explosives of the nitroaromatic class. The existence of a built-in fluorophore in calixarenes was discussed, as well as the use of extrinsic aromatic fluorophores for a variety of purposes, including the measurement of temperature above 100°C.

Future lines of research include, not only the development of homooxacalixarenes with pendant groups increasing the selectivity and affinity for specific analytes, but also modifications allowing the use in aqueous and biological media. In the last case, given the known transmission spectral window, red and NIR emitting fluorophores will be particularly advantageous.
